# Effect of Lime, Humic Acid and Moisture Regime on the Availability of Zinc in Alfisol

**DOI:** 10.1100/tsw.2007.192

**Published:** 2007-08-17

**Authors:** Sushanta Kumar Naik, Dilip Kumar Das

**Affiliations:** ^1^ National Research Centre for Orchids, Pakyong-737106, Sikkim, India; ^2^ Department of Agricultural Chemistry and Soil Science, Faculty of Agriculture, Bidhan Chandra Krishi Viswavidyalaya, Mohanpur, Nadia-741252, West Bengal, India

**Keywords:** Alfisol, Humic acid, Lime, Moisture regime, Zinc availability

## Abstract

Lime and humic acid application can play an important role in the availability of zinc in paddy soils. We conducted laboratory incubation experiments on a rice growing soil (Alfisol) to determine the effect of lime, humic acid and different moisture regimes on the availability of Zn. Addition of half doses of liming material (powdered lime stone) recorded highest values of DTPA-Zn followed by no lime and 100% of lime requirement throughout the incubation period. With the progress of incubation, DTPA-Zn increased slightly during the first week and then decreased thereafter. The highest DTPA-extractable Zn content of 2.85 mg/kg was found in the treatment Zn_10_ L_1/2_ at 7 days of incubation, showing 17.3 % increase in DTPA-Zn content over its corresponding treatment of Zn alone (Zn_10_L_0_). The DTPA-Zn concentration increased with the application of humic acid compared with no humic acid throughout 35 days of the incubation period and the peak value obtained was 3.12 mg/kg in the treatment Zn10 HA2 at 14 days after incubation, showing 50 % increase in Zn content over its corresponding treatment of Zn alone (Zn_10_HA_0_). The application of 0.2% humic acid compared with 0.1% resulted in greater increase in DTPA-Zn concentration in soil application. During the 35 days of incubation, highest values of DTPA-Zn were recorded in soil maintained at saturated compared to water logged conditions. However, under alternate wetting and drying condition the DTPA-Zn content gradually decreased up to 21 days and thereafter increased slowly.

## INTRODUCTION

Zinc (Zn) undergoes transformation in soils by various mechanisms i.e. sorption by clays, hydrous oxides, organic matter etc., which affect its availability of Zn in soils. Desorption of Zn into soil solution is controlled by the energy with which it is adsorbed onto the soil colloidal surfaces. This in turn depends on the soil characteristics particularly pH, cation exchange capacity (CEC), the nature and content of the clay, oxides of Fe, Al and Mn, and $CaCO_3$[1]. The problem of Zn deficiency in flooded rice soils was believed to be associated with high pH or high amounts of $CaCO_3$ and partly due to the intense reduction of soil causing increased concentration of ferrous iron ($Fe^{2+}$) and manganous manganese ($Mn^{2+}$) in soil[2].

When soils are submerged the concentrations of most nutrient elements in the soils increases, but this is not true for Zn. In acid soils Zn availability decreases after flooding owing to an increase in pH and the precipitation of $Zn(OH)_2$. However, if an alkali soil is submerged, the pH of the soil will decrease and as a result the solubility of Zn will increase. The availability of Zn has been found to be decreased with an increase in the amount of lime to the soil. Zn has also been found to be adsorbed less strongly on calcite ($CaCO_3$) than magnesite and dolomite. Alfisol was characterized by grey to brown surface horizon, medium to high base supply, and subsurface horizons of clay accumulation, usually moist but may be dry during warm season. An Alfisol may also have fragipan, duripan, natric horizon, petrocalcic horizon, and plinthite. Some of the red and lateritic soils of India have been classified as Alfisol.

Soil organic matter mainly consists of humic and fulvic acids are called humin materials[3,4]. It was reported that humic acid affects chemical properties of soils due to carboxyl (-COOH) and phenolic (-OH) groups[5,6,7]. The release and uptake of the $Zn^{2+}$, being the important micronutrient element in soil, is often regulated by the humic or fulvic substances present in the soil. Zn forms stable complexes with soil organic matter, where humic acid and fulvic acid fractions play an important role in the adsorption processes. Randhawa and Broadbent[8] found that three or more types of sites were involved in the retention of Zn by humic acids. The least stable fraction, which accounted for most of the adsorbed Zn, was believed to be associated with phenolic OH and COOH groups having pKa values between 2.8 and 4.4. The more stable fraction was assigned to strongly acidic COOH groups with lower pKa values (< 2.0). Although the strongly bound Zn represented less than 1% of the total retained, the sites responsible were believed to be of great importance because small quantities of Zn would be adsorbed preferentially in the most stable forms.

Mandal et al.[9] observed that desorption of Zn in Alfisols was higher under alternate wetting and drying (67.1%) followed by flooded-dried (61.4%) and preflooding (47.3%) moisture regimes. The variation in Zn-desorption among soils and moisture treatments is the changes in soil pH, Fe-oxides, bonding energy constants, and free energies for Zn adsorption. Variation in the soil's physico-chemical properties are largely controlled by different soil moisture regimes as soil moisture regimes control the restricted entry of atmospheric oxygen or escaping tendency of oxygen from soils to the atmosphere. Therefore, different soil moisture regimes bring about varying changes in soil physico-chemical and electrochemical properties such as pH, Eh, electrical conductivity, $CaCO_3$ content, and amorphous and crystalline oxides of Fe and Mn, which are further accentuated by application of starch or manures[9].

Since problem of Zn deficiency persists in the soil under different moisture regime conditions and the requirement of Zn to the growing plant is essential for balanced nutrition. Therefore, an attempt has been made to study the effect of lime, humic acid, and moisture regime on the availability of Zn.

## EXPERIMENTAL METHODS

The soil sample was collected from the farmer's paddy field at Jhargram (Alfisol) in the West Midnapur district of West Bengal state, India during the year 2005. These were air dried, powdered and sieved through an 80 mesh sieve and analyzed for its different chemical properties following the standard methods of Jackson[10] and Black et al.[11]. The chemical properties of the experimental soil were: pH 5.6, EC 0.07 dS/m, CEC 16.21 cmol ($p^+$)/kg, Organic carbon 2.9 g/kg, DTPA extractable Zn 1.58 mg/kg, with 60% sand, 17% silt, and 23% clay (loam texture).

### Soil Incubation With Lime

Triplicate samples of 10g each of the air-dried soils were taken in a series of incubation tubes. Three levels of Zn at 0 ($Zn_0$), 5 ($Zn_5$) and 10 ($Zn_{10}$) mg/kg as $ZnSO_4.7H_2O$ and three levels of lime ($CaCO_3$) i.e., 0 ($L_0$), ½ lime requirement ($L_{1/2}$) and full lime requirement ($L_1$) were added to the experimental soil under saturated conditions. The pure $CaCO_3$ required for 10g soil corresponding to ½ lime requirement and full lime requirement were 15 and 30 mg, respectively. The incubation tubes were laid down in completely randomized design (CRD) with factorial concept. The treatment combinations were 9 with three replications. Lime requirement (LR) of acid soil was determined by using the buffer method of Shoemaker et al.[12].

## Soil Incubation With Humic Acid

Humic acid was extracted from FYM (Spell out when you mention it first time Farmyard manure (FYM) solution by repeated treatments with 0.1N NaOH solution at room temperature: FYM ratio of 10:1 and separated on the basis of their solubility in inorganic acids according to the procedure outlined by Stevenson[13]. The $E_4/E_6$ ratios were determined according to the procedure described by Chen et al.[14]. Carbon and Hydrogen were estimated by CH analyzer, nitrogen by micro-kjeldahl method and oxygen content was calculated by difference. Total acidity was determined by Ba $(OH)_2$ titration and carboxyl by calcium acetate method[15].

Phenolic hydroxyls were obtained by difference between total acidity and carboxyl groups. The ash content was obtained by burning a certain amount of humic acid in an oven at $900^0$C for six hour. The characteristics of humic acid extracted from FYM were given in Table 1.Triplicate samples of 10g each of the air-dried soils were taken in a series of incubation tubes. Three levels of Zn at 0, 5, and 10 mg/kg as $ZnSO_4.7H_2O$ and three levels of humic acid i.e., 0 ($HA_0$), 0.1 ($HA_1$) and 0.2 ($HA_2$) % by weight were added to the experimental soil under saturated condition. The incubation tubes were laid down in completely randomized design (CRD) with factorial concept. The treatment combinations were 9 with three replications.

**Table 1**
**Characteristics of Humic Acid Extracted From Farmyard Manure (FYM)**

| Properties | Particulars |
|---|---|
| C (%) | 53.6 |
| H (%) | 4.8 |
| N (%) | 2.7 |
| O (%) | 37.2 |
| Ash (%) | 1.6 |
| $E_4/E_6$ | 3.2 |
| Total acidity (cmol/kg) | 570 |
| Carboxyl (cmol/kg) | 340 |
| Phenolic-OH ((cmol/kg) | 230 |

## Soil Incubation Under Different Moisture Regimes

Triplicate samples of 10g each of the air dried soils were taken in a series of incubation tubes and three levels of Zn at 0, 5 and 10 mg/kg as $ZnSO_4.7H_2O$ was added under three moisture regime conditions viz., saturated ($M_1$), waterlogged ($M_2$) and alternate wetting and drying ($M_3$). The incubation tubes were laid down in completely randomized design (CRD) with factorial concept. The treatment combinations were 9 with three replications. The saturated condition was maintained based on maximum water holding capacity of the soil. Waterlogged condition (depth above the soil surface 2±0.5 cm) was maintained in corresponding treatment combinations throughout the period of investigation by applying double distilled water. The alternate wetting and drying condition was maintained by just saturating the soil followed by drying to field capacity.

In all the incubation experiments, the soils were allowed to incubate at room temperature $(25\pm2)^0$C for a period of 0, 7, 14, 21, 28, and 35 days after moistening to appropriate moisture level. Loss of moisture due to evaporation was replenished by the addition of double distilled water on every alternate day by difference in weight. 0.005 M DTPA solutions were used to extract the available pool of Zn from the soil sample of all the incubated experiments using Lindsay and Norvell[16]. After extraction, Zn was determined with the help of an Atomic Absorption Spectrophotometer (Perkin Elmer Model: A Analyst 100). The relevant data were statistically analyzed for Duncan's Multiple Range Test (DMRT) using the statistical computer programme MSTAT, version 5 (New Delhi, India).

## RESULTS AND DISCUSSION

### Effect of Lime

As can be seen in Table 2 the application of lime at half LR in combination with Zn to Alfisol significantly increased the DTPA extractable Zn over no lime and Zn ($Zn_0L_0$) treated soil throughout the incubation period of 35 days. The interaction effect between Zn and lime was significant among the different treatments. It was observed that the DTPA extractable Zn slightly increased during first week of incubation and thereafter gradually decreased with the progress of incubation in all the treatments under study. The increase in DTPA extractable Zn in the soil during first week of incubation may be due to different chemical and biochemical changes occurring, as a result of moistening the partially air dried soil[17].

The DTPA extractable Zn content were found to be highest in the treatment where Zn was applied at its highest level of 10.0 mg/kg combined with half LR of the soil ($Zn_{10}$ $L_{1/2}$) at all days of incubation. At 7 days of incubation, the DTPA extractable Zn recorded the highest (2.85 mg/kg), when Zn was applied at 10.0 mg/kg in combination with half LR of soil ($Zn_{10}$ $L_{1/2}$). Lime applied at half LR in presence or absence of Zn to the Alfisol found to be significantly better in maintaining the DTPA extractable Zn in the soil compared to no lime and full LR of soil at various levels of Zn throughout the incubation period.

Comparing the mean effect of applied Zn on the availability of DTPA extractable Zn in lime treated Alfisol; it was observed that application of Zn from 0-10 mg/kg significantly increased the DTPA extractable Zn over no Zn throughout the incubation period (Table 2). The DTPA extractable Zn was found to be highest when Zn was applied at 10.0 mg/kg and followed the trend, $Zn_{10} > Zn_5 > Zn_0$. The DTPA-extractable Zn initially increased up to 7 days and thereafter gradually decreased. The percent decrease in the DTPA extractable Zn from 0 to 35 days after incubation was 101.3, 103.61 and 122.33 % at 0, 5 and 10 mg/kg of applied zinc, respectively. There was a rapid fall in the DTPA extractable Zn during early period of incubation (up to 21 days) and thereafter it decreased slowly. The sharp decrease may be due to the adsorption of Zn on hydrous oxides of Fe and Mn in the soil[18].

Similarly the mean effect of lime on the availability of Zn showed that lime at half LR of soil recorded more DTPA extractable Zn compared to no lime and full LR of soil at all days of incubation and followed the trend as $L_{1/2} > L_0 > L_1$ (Table 2). However, the DTPA extractable Zn in no lime ($L_0$) and full LR ($L_1$) treatments were at par throughout the incubation, except at 0 days. The DTPA extractable Zn at various levels of applied lime increased in the first week of incubation and thereafter gradually decreased. The percent decrease in DTPA extractable Zn from 0 to 35 days after incubation was 123.75, 100.94 and 107.69% at 0, half LR and full LR of soil, respectively. The increase in the DTPA extractable Zn following addition of half LR to soil may be due to the slight increase in pH of the soil resulting in adsorption of Zn on organic matter where it is selectively retained as a low molecular weight organic complex [19]. However with the increase in lime to Alfisol at full LR, the DTPA extractable Zn decreased compared to no lime application, which might be due to the formation of insoluble Zn or precipitation of Zn with $CaCO_3$[2].

**Table 2**
**Effect of Lime on the Availability of The DTPA Extractable Zinc (mg/kg) in an Alfisol at Saturated Condition**

| Treatments | DTPA extractable zinc (mg/kg) | | | | | |
|---|---|---|---|---|---|---|
| | Days after Incubation | | | | | |
| | 0 | 7 | 14 | 21 | 28 | 35 |
| **Zinc levels** | | | | | | |
| $Zn_0$ | 1.55$^{C†}$ | 1.64$^{C}$ | 1.41$^{C}$ | 1.07$^{C}$ | 0.85$^{C}$ | 0.77$^{B}$ |
| $Zn_5$ | 1.69$^{B}$ | 1.80$^{B}$ | 1.54$^{B}$ | 1.19$^{B}$ | 0.98$^{B}$ | 0.83$^{B}$ |
| $Zn_{10}$ | 2.29$^{A}$ | 2.46$^{A}$ | 2.13$^{A}$ | 1.74$^{A}$ | 1.39$^{A}$ | 1.03$^{A}$ |
| **LR levels** | | | | | | |
| $L_0$ | 1.79$^{B}$ | 1.87$^{B}$ | 1.62$^{B}$ | 1.24$^{B}$ | 0.98$^{B}$ | 0.80$^{B}$ |
| $L_{1/2}$ | 2.13$^{A}$ | 2.27$^{A}$ | 1.93$^{A}$ | 1.58$^{A}$ | 1.29$^{A}$ | 1.06$^{A}$ |
| $L_1$ | 1.62$^{C}$ | 1.77$^{B}$ | 1.53$^{B}$ | 1.18$^{B}$ | 0.96$^{B}$ | 0.78$^{B}$ |
| **Interaction** | | | | | | |
| $Zn_0$ $L_0$ | 1.42$^{e}$ | 1.52$^{e}$ | 1.35$^{d}$ | 0.97$^{e}$ | 0.77$^{e}$ | 0.70$^{c}$ |
| $Zn_0$ $L_{1/2}$ | 1.80$^{cd}$ | 1.86$^{d}$ | 1.52$^{d}$ | 1.24$^{d}$ | 1.01$^{c}$ | 0.90$^{b}$ |
| $Zn_0$ $L_1$ | 1.43$^{e}$ | 1.55$^{e}$ | 1.38$^{d}$ | 0.99$^{e}$ | 0.79$^{de}$ | 0.71$^{c}$ |
| $Zn_5$ $L_0$ | 1.61$^{de}$ | 1.66$^{e}$ | 1.44$^{d}$ | 1.11$^{de}$ | 0.88$^{d}$ | 0.76$^{c}$ |
| $Zn_5$ $L_{1/2}$ | 1.92$^{c}$ | 2.10$^{c}$ | 1.77$^{c}$ | 1.43$^{c}$ | 1.26$^{b}$ | 1.01$^{b}$ |
| $Zn_5$ $L_1$ | 1.54$^{e}$ | 1.64$^{e}$ | 1.41$^{d}$ | 1.05$^{e}$ | 0.82$^{de}$ | 0.74$^{c}$ |
| $Zn_{10}$ $L_0$ | 2.33$^{b}$ | 2.43$^{b}$ | 2.08$^{b}$ | 1.65$^{b}$ | 1.30$^{b}$ | 0.93$^{b}$ |
| $Zn_{10}$ $L_{1/2}$ | 2.65$^{a}$ | 2.85$^{a}$ | 2.51$^{a}$ | 2.08$^{a}$ | 1.60$^{a}$ | 1.27$^{a}$ |
| $Zn_{10}$ $L_1$ | 1.88$^{c}$ | 2.11$^{c}$ | 1.82$^{c}$ | 1.50$^{c}$ | 1.28$^{b}$ | 0.90$^{b}$ |

$Zn_0$= no zinc; $Zn_5$= Zn at 5 mg/kg as $ZnSO_4.7H_2O$; $Zn_{10}$= Zn at 10 mg/kg as $ZnSO_4.7H_2O$; $L_0$= no lime; $L_{1/2}$=½ lime requirement; $L_1$= full lime requirement; LR= Lime requirement. †Within a column, data followed by the same letter are not significantly different at the 0.05 level of probability by DMRT.

## Effect of Humic Acid

The perusal of the data in table 3 shows that the DTPA extractable Zn content of soil significantly increased following application of different levels of Zn and humic acid either in sole or in combination at all days of incubation. The interaction effect between Zn and humic acid was significant among the different treatments. The study showed that the treatments receiving only Zn ($Zn_0$ $HA_0$, $Zn_5$ $HA_0$, $Zn_{10}$ $HA_0$), DTPA extractable Zn contents increased up to 7 days of incubation and thereafter decreased. However, the treatments receiving humic acid alone ($Zn_0$ $HA_1$, $Zn_0$ $HA_2$) or in combination with Zn ($Zn_5$ $HA_1$, $Zn_5$ $HA_2$, $Zn_{10}$ $HA_1$, $Zn_{10}$ $HA_2$), the DTPA extractable Zn content increased slowly up to 14 days of incubation and thereafter gradually decreased.

The velocity and magnitude of increase in the DTPA-Zn content to reach the peak value varied. In all the cases, the peak value obtained was 3.12 mg/kg in the treatment $Zn_{10}$ $HA_2$ (Zn at 10 mg/kg as $ZnSO_4.7H_2O$ + humic acid at 0.2% by weight of soil) at 14 days after incubation followed by a gradual decrease throughout the incubation period. The increased during initial period of 14 days might be due to the coating of Fe-oxides surfaces by the humic acid, thereby decreasing their adsorptivity for metals and thus metals bound under such conditions might be desorbed readily. Humic acid forms complex with Fe and Mn compound in earlier stage of incubation and results in less specific surface area for adsorption of Zn and the results are in conformity with the earlier findings of Garcia et al.[20].

**Table 3**
**Effect of Humic Acid (HA) on the Availability of The DTPA Extractable Zinc (mg/kg) in an Alfisol at Saturated Condition**

| Treatments | DTPA extractable zinc (mg/kg) | | | | | |
|---|---|---|---|---|---|---|
| | Days after Incubation | | | | | |
| | 0 | 7 | 14 | 21 | 28 | 35 |
| **Zinc levels** | | | | | | |
| $Zn_0$ | $1.65^{C†}$ | $1.75^{C}$ | $1.78^{C}$ | $1.30^{C}$ | $0.95^{C}$ | $0.79^{C}$ |
| $Zn_5$ | $1.99^{B}$ | $2.12^{B}$ | $2.16^{B}$ | $1.76^{B}$ | $1.49^{B}$ | $1.20^{B}$ |
| $Zn_{10}$ | $2.68^{A}$ | $2.77^{A}$ | $2.72^{A}$ | $2.27^{A}$ | $1.79^{A}$ | $1.32^{A}$ |
| **HA levels** | | | | | | |
| $HA_0$ | $1.79^{C}$ | $1.87^{C}$ | $1.62^{C}$ | $1.24^{C}$ | $0.98^{C}$ | $0.80^{C}$ |
| $HA_1$ | $2.12^{B}$ | $2.26^{B}$ | $2.38^{B}$ | $1.95^{B}$ | $1.46^{B}$ | $1.17^{B}$ |
| $HA_2$ | $2.40^{A}$ | $2.51^{A}$ | $2.66^{A}$ | $2.15^{A}$ | $1.79^{A}$ | $1.35^{A}$ |
| **Interaction** | | | | | | |
| $Zn_0 HA_0$ | $1.42^{f}$ | $1.52^{d}$ | $1.35^{f}$ | $0.97^{f}$ | $0.77^{g}$ | $0.70^{e}$ |
| $Zn_0 HA_1$ | $1.55^{e}$ | $1.64^{d}$ | $1.76^{e}$ | $1.41^{e}$ | $0.99^{ef}$ | $0.79^{de}$ |
| $Zn_0 HA_2$ | $1.97^{d}$ | $2.10^{c}$ | $2.22^{d}$ | $1.53^{de}$ | $1.10^{e}$ | $0.88^{de}$ |
| $Zn_5 HA_0$ | $1.61^{e}$ | $1.66^{d}$ | $1.42^{f}$ | $1.10^{f}$ | $0.88^{fg}$ | $0.76^{de}$ |
| $Zn_5 HA_1$ | $2.07^{d}$ | $2.28^{bc}$ | $2.43^{c}$ | $1.99^{c}$ | $1.61^{c}$ | $1.31^{c}$ |
| $Zn_5 HA_2$ | $2.28^{c}$ | $2.41^{b}$ | $2.64^{b}$ | $2.20^{c}$ | $1.97^{b}$ | $1.54^{ab}$ |
| $Zn_{10} HA_0$ | $2.33^{c}$ | $2.43^{b}$ | $2.08^{d}$ | $1.65^{d}$ | $1.30^{d}$ | $0.93^{d}$ |
| $Zn_{10} HA_1$ | $2.75^{b}$ | $2.87^{a}$ | $2.95^{a}$ | $2.44^{b}$ | $1.77^{c}$ | $1.41^{bc}$ |
| $Zn_{10} HA_2$ | $2.96^{a}$ | $3.03^{a}$ | $3.12^{a}$ | $2.73^{a}$ | $2.29^{a}$ | $1.63^{a}$ |

$Zn_0$= no zinc; $Zn_5$= Zn at 5 mg/kg as $ZnSO_4.7H_2O$; $Zn_{10}$= Zn at 10 mg/kg as $ZnSO_4.7H_2O$; $HA_0$= no humic acid; $HA_1$= humic acid at 0.10% by weight of soil; $HA_2$= humic acid at 0.20% by weight of soil. †Within a column, data followed by the same letter are not significantly different at the 0.05 level of probability by DMRT.

The mean effect of applied Zn on the availability of the DTPA extractable Zn in humic acid treated Alfisol showed that the extractable Zn varied significantly among the various levels of Zn from 0-10 mg/kg throughout the incubation period (Table 3). The DTPA extractable Zn attained its peak value when Zn was applied at the highest level of 10.0 mg/kg ($Zn_{10}$) at all days of incubation and was significantly higher than the other two levels of applied Zn ($Zn_5$ and $Zn_0$). There is a slight increase in DTPA extractable Zn from 0 to 14 days after incubation and thereafter decreased sharply to 35 days at all levels of applied Zn. The percent decrease in the DTPA extractable Zn from 0 to 35 days of incubation was 108.8, 65.83 and 103.03 % at 0, 5 and 10 mg/kg of applied Zn, respectively.

The mean effect of humic acid on the availability of DTPA extractable Zn in the Alfisol showed that the application of humic acid (0.1-0.2% by weight of the soil) significantly increased the extractable Zn over no humic acid application (Table 3). The DTPA extractable Zn gradually increased from 0 to 14 days after incubation and thereafter decreased to 35 days at all levels of humic acid application. The results are in conformity with the findings of Natesan et al.[21] who reported that the positive effect of humic acid in increasing the availability of micronutrients may be due to its priming effect to increase water soluble amounts of micronutrients, which led to chelation and subsequent release of micronutrients.

The peak value of DTPA extractable Zn was found the highest level of humic acid (0.2% by weight of soil) throughout the incubation period and followed the trend, $HA_2$ > $HA_1$ > $HA_0$. Ong and Busque[22], in a study observed that newly formed humic substances are more mobile and can act as solubilizers of mineral matter, thereby increasing the availability of micronutrients to plants. The percent decrease in DTPA extractable Zn from 0 to 35 days after incubation was 123.75, 81.20 and 77.77 % at 0, 0.1 and 0.2% of humic acid by weight of soil, respectively.

## Effect of Moisture Regime

The DTPA extractable Zn was significantly influenced by different levels of Zn applied under different moisture regimes in the Alfisol studied (Table 4). The interaction between Zn and moisture regime was significant among the different treatments. The DTPA extractable Zn increased in the first week of incubation and thereafter gradually decreased throughout the incubation period under different moisture regimes. The highest DTPA extractable Zn of 2.43 mg/kg was recorded when 10.0 mg/kg of Zn was applied to the Alfisol under saturated condition ($Zn_{10}$ $M_1$), after 7 days of incubation. Zn applied under saturated condition recorded significantly higher DTPA extractable Zn over that of soil maintained at waterlogged condition and was at par with the soil maintained at alternate wetting and drying up to 21 days of incubation. These results find support from Mandal et al.[9] who reported that the higher amount of DTPA extractable Zn under flooded-dried and alternate wetting and drying compared to pre-flooding condition at all levels of applied Zn and could be attributed to the lower values of bonding energy constants and free energy changes for Zn in the former two moisture regimes. The low content of DTPA extractable Zn observed under waterlogged compared to saturated and alternate wetting and drying conditions at each incubation period may also be due to the increased formation of $Fe^{2+}$ and $Mn^{2+}$ due to reduction of its higher valent oxides and $Zn^{2+}$ gets adsorbed on the hydrous oxides of iron. These findings are in accordance with those of Mandal and Mandal[23]. Singh et al.[24] reported that the percent recovery of applied Zn as water soluble and exchangeable Zn significantly higher under alternate submergence and saturation than continuous submergence condition.

The mean effect of the applied Zn on the DTPA extractable Zn under different moisture regimes was found to be significantly higher with the addition of Zn from 0-10 mg/kg over no Zn application, throughout the incubation period (Table 4). The highest value of extractable Zn was recorded at the highest level of Zn (10.0 mg/kg) and followed the trend, $Zn_{10}$ > $Zn_5$ > $Zn_0$. However with the progress of incubation, the DTPA extractable Zn gradually decreased from 7 to 35 days. The decrease in the DTPA extractable Zn has been attributed to the adsorption of Zn on the poorly crystalline sesquioxide surfaces which has a high affinity for zinc [23]. The percent decrease in the DTPA extractable Zn from 0 to 35 days after incubation was 126.22, 68.88 and 95.53 % at 0, 5 and 10 mg/kg of applied Zn, respectively.

However the mean effect of different moisture regimes, i.e. saturation, water logging, and alternate wetting and drying, significantly decreased the DTPA extractable Zn throughout incubation, except the first week of incubation where there is a slight increase in the DTPA extractable Zn from 0 to 7 days period (Table 4). When the soil was maintained at saturated condition, the DTPA extractable Zn content was significantly higher than that of soil maintained at water logging condition. However, the soil maintained at alternate wetting and drying, the DTPA extractable Zn content decreased gradually up to 21 days and thereafter increased slowly. This increase is likely due to the reactivity of adsorbing complexes, such as like Fe and Mn-oxides[9] and decrease in bonding energy constant and free energy change for Zn adsorption[25]. The increase in the DTPA extractable Zn may also be attributed to the release of Zn from insoluble zinc minerals, such as franklinite ($ZnFe_2O_4$), following drying condition in the soil[26].

**Table 4**
**Effect of Different Moisture Regimes (MR) on the Availability of DTPA Extractable Zinc (mg/kg) in the Alfisol Studied**

| Treatments | DTPA-extractable zinc (mg/kg) | | | | | |
|---|---|---|---|---|---|---|
| | Days after Incubation | | | | | |
| | 0 | 7 | 14 | 21 | 28 | 35 |
| **Zinc levels** | | | | | | |
| $Zn_0$ | $1.38^{C\dagger}$ | $1.49^{C}$ | $1.24^{C}$ | $0.78^{C}$ | $0.70^{C}$ | $0.61^{C}$ |
| $Zn_5$ | $1.52^{B}$ | $1.61^{B}$ | $1.38^{B}$ | $1.06^{B}$ | $0.93^{B}$ | $0.90^{B}$ |
| $Zn_{10}$ | $2.19^{A}$ | $2.38^{A}$ | $2.02^{A}$ | $1.60^{A}$ | $1.34^{A}$ | $1.12^{A}$ |
| **MR levels** | | | | | | |
| $M_1$ | $1.79^{A}$ | $1.87^{A}$ | $1.62^{A}$ | $1.24^{A}$ | $0.98^{B}$ | $0.80^{B}$ |
| $M_2$ | $1.63^{B}$ | $1.77^{B}$ | $1.45^{B}$ | $1.07^{B}$ | $0.82^{C}$ | $0.57^{C}$ |
| $M_3$ | $1.68^{B}$ | $1.83^{A}$ | $1.57^{A}$ | $1.13^{B}$ | $1.17^{A}$ | $1.27^{A}$ |
| **Interaction** | | | | | | |
| $Zn_0 M_1$ | $1.42^{de}$ | $1.52^{de}$ | $1.35^{cd}$ | $0.97^{c}$ | $0.77^{d}$ | $0.70^{d}$ |
| $Zn_0 M_2$ | $1.34^{e}$ | $1.46^{e}$ | $1.06^{e}$ | $0.66^{d}$ | $0.57^{e}$ | $0.32^{e}$ |
| $Zn_0 M_3$ | $1.39^{de}$ | $1.49^{e}$ | $1.31^{d}$ | $0.73^{d}$ | $0.76^{d}$ | $0.82^{cd}$ |
| $Zn_5 M_1$ | $1.61^{c}$ | $1.66^{c}$ | $1.44^{c}$ | $1.10^{b}$ | $0.88^{d}$ | $0.76^{cd}$ |
| $Zn_5 M_2$ | $1.44^{de}$ | $1.55^{de}$ | $1.31^{d}$ | $1.01^{bc}$ | $0.79^{d}$ | $0.63^{d}$ |
| $Zn_5 M_3$ | $1.50^{cd}$ | $1.61^{cd}$ | $1.39^{cd}$ | $1.06^{bc}$ | $1.12^{c}$ | $1.31^{b}$ |
| $Zn_{10} M_1$ | $2.33^{a}$ | $2.43^{a}$ | $2.08^{a}$ | $1.65^{a}$ | $1.30^{b}$ | $0.93^{c}$ |
| $Zn_{10} M_2$ | $2.10^{b}$ | $2.31^{b}$ | $1.97^{b}$ | $1.54^{a}$ | $1.09^{c}$ | $0.77^{cd}$ |
| $Zn_{10} M_3$ | $2.14^{b}$ | $2.40^{ab}$ | $2.01^{ab}$ | $1.61^{a}$ | $1.64^{a}$ | $1.67^{a}$ |

$Zn_0$= no zinc; $Zn_5$= Zn at 5 mg/kg as $ZnSO_4.7H_2O$; $Zn_{10}$= Zn at 10 mg/kg as $ZnSO_4.7H_2O$; $M_1$= Saturated condition; $M_2$=Waterlogged condition; $M_3$= Alternate wetting and drying; LR= Lime requirement. †Within a column, data followed by the same letter are not significantly different at the 0.05 level of probability by DMRT.

## CONCLUSIONS

The results explore the effect of lime, humic acid and different moisture regimes on the availability of Zn in the Alfisol studied. The DTPA extractable Zn under ½ LR was found appreciably higher followed by no lime and full LR at saturated condition. Furthermore, application of humic acid enhance the availability of Zn in 14 days of incubation and higher value of the DTPA extractable Zn was obtained throughout the incubation period compared to no humic acid application with saturated condition. Among the different moisture regimes, saturated and alternate wetting and drying conditions proved to be better in enhancing the availability of Zn throughout the incubation. From this experiment it is concluded that the ½ LR and humic acid application have favorable effect on the Zn availability at saturated condition. Soil kept under saturated and alternate wetting and drying conditions have greater impact on Zn availability.
